# An age-period-cohort analysis of mortality rates for stomach, colorectal, liver, and lung cancer among prefectures in Japan, 1999–2018

**DOI:** 10.1186/s12199-020-00922-0

**Published:** 2020-12-05

**Authors:** Tasuku Okui

**Affiliations:** Medical Information Center, Kyushu University Hospital, Maidashi 3-1-1 Higashi-ku, Fukuoka City, Fukuoka Prefecture 812-8582 Japan

**Keywords:** Cohort effect, Colorectal cancer, Japan, Liver cancer, Lung cancer, Mortality, Stomach cancer

## Abstract

**Background:**

Although change in the birth cohort effect on cancer mortality rates is known to be highly associated with the decreasing rates of age-standardized cancer mortality rates in Japan, the differences in the trends of cohort effect for representative cancer types among the prefectures remain unknown. This study aimed to investigate the differences in the decreasing rate of cohort effects among the prefectures for representative cancer types using age-period-cohort (APC) analysis.

**Methods:**

Data on stomach, colorectal, liver, and lung cancer mortality for each prefecture and the population data from 1999 to 2018 were obtained from the Vital Statistics in Japan. Mortality data for individuals aged 50 to 79 years grouped in 5-year increments were used, and corresponding birth cohorts born 1920–1924 through 1964–1978 were used for analysis. We estimated the effects of age, period, and cohort on each type of mortality rate for each prefecture by sex. Then, we calculated the decreasing rates of cohort effects for each prefecture. We also calculated the mortality rate ratio of each prefecture compared with all of Japan for cohorts using the estimates.

**Results:**

As a result of APC analysis, we found that the decreasing rates of period effects were small and that there was a little difference in the decreasing rates among prefectures for all types of cancer among both sexes. On the other hand, there was a large difference in the decreasing rates of cohort effects for stomach and liver cancer mortality rates among prefectures, particularly for men. For men, the decreasing rates of cohort effects in cohorts born between 1920–1924 and 1964–1978 varied among prefectures, ranging from 4.1 to 84.0% for stomach cancer and from 20.2 to 92.4% for liver cancers, respectively. On the other hand, the differences in the decreasing rates of cohort effects among prefectures for colorectal and lung cancer were relatively smaller.

**Conclusions:**

The decreasing rates of cohort effects for stomach and liver cancer varied widely among prefectures. It is possible that this will influence cancer mortality rates in each prefecture in the future.

## Background

Cancer is the primary cause of mortality in Japan, and the mortality rate continues to increase along with by the aging of the population [1]. Although age-standardized mortality rates of all cancer sites have been decreasing in recent years, this decrease in rates is different depending on the type of cancer [1]. Stomach, colorectal, liver, pancreatic, and lung cancer were the top 5 causes of cancer mortality in 2018 [1]. It is known that age-standardized mortality rates of stomach and liver cancers in particular have decreased in recent years and that these decreases have contributed a decrease in age-standardized mortality rates of all-sites cancer in Japan [2]. The decreasing rates for colorectal and lung cancer were relatively smaller. The rate for pancreatic cancer, on the other hand, has increased [3]. Social burdens associated with cancer mortality rates are large in Japan, and further prevention of cancer is necessary.

It is known that cancer mortality rates and any decrease in these rates vary depending on region [4]. There are disparities in the decreasing rates among prefectures, or administrative districts, in Japan. In addition, trends of decreasing rates for prefectures differ depending on cancer type [4], possibly because the trend of the prevalence of each of the risk factor was different among prefectures. Moreover, it is known that cancer mortality rates have been decreasing in cohorts by sex, particularly for men, and that the decreasing rates of the cohort effect have a large impact on the decreasing mortality rate of each type of cancer [5]. Although it is believed that the decreasing rates of the cohort effect are different among prefectures, the difference has not been investigated for most cancer types. Age-period-cohort (APC) analysis is often used as a method for identifying cohort effect on disease mortality [6]. By using APC analysis, we can distinguish age, period, and cohort effect for the change in mortality rates. Although many studies using APC analysis for each type of cancer have been conducted in Japan [7–9], APC analyses investigating the differences in each effect among prefectures have been conducted only for all-sites and pancreatic cancer [10, 11]. By assessing the cohort effects on mortality rates of each cancer type, we can better understand the reason for the change in the cancer mortality rates for each prefecture and can also assess which cohorts need further preventive measures for each type of cancer. In this study, we analyzed the differences in the trends of stomach, colorectal, liver, and lung cancer mortality rates in Japan among prefectures using an APC analysis and also revealed the differences in cohort effects among prefectures.

## Methods

We analyzed cancer mortality data obtained from the Vital Statistics in Japan from 1999 to 2018 [1]. Mortality data since 1999 for each type of cancer for each prefecture are publicly available online. The International Classification of Diseases (10th Revision) codes corresponding to each type of cancer are as follows: stomach, C16; colorectal, C18–20; liver, C22, and lung, C33–34. The populations of the prefectures for each age group, sex, and year were also extracted from the Vital Statistics in Japan [12]. Individuals aged 50–54 years and 75–79 years, in 5-year increments, were included in our analysis. Although data for individuals aged 0–79 years are publicly available, the cancer mortality data for younger people are sparse for each prefecture. Therefore, we used data for those aged 50 years or older. A cohort was defined for each age group for each year. A total of 45 cohorts were defined and used for analysis. Then, the age group 75–79 years at 1999 (i.e., those who were born in 1920–1924) was the oldest birth cohort in the APC analysis. Through a 1-year shift starting from the oldest cohort, the age group 50–54 years in 2018 (i.e., those who were born in 1964–1968) was the most recently born cohort.

For the statistical analysis, we calculated age-standardized mortality rates in 1999 and 2018 for each type of cancer and prefecture to assess the change of the mortality rates during the analyzed periods. The population ratio of the total population in 1999 was used as the reference population for the calculation of the age-standardized mortality rates. We used the Bayesian APC model [6] in our study according to the following equation: Let *y*_*ij*_ be the cancer mortality of a prefecture for the age group *i* (1, …, *I*) in year *j* (1, …, *J*). In the model, *y*_*ij*_ are assumed to follow the following Poisson distribution whose mean is *λ*_*ij*_:

$$ {y}_{ij}\sim \mathrm{Poisson}\left({\lambda}_{ij}\right), $$

$$ \log \left({\lambda}_{ij}\right)=\delta +{\alpha}_i+{\beta}_j+{\gamma}_k+{z}_{ij}+\log \left({n}_{ij}\right) $$

where *δ* is the intercept, *α*_*i*_ are the effects of age groups, *β*_*j*_ are period effects, *γ*_*k*_ (*k* = 1, …, *K*) are cohort effects, *z*_*ij*_ are random effects that are defined for each year and age group, and *n*_*ij*_ are the corresponding population. *I*, *J*, and *K* are the total number of time points for each effect, and *I* = 6, *J* = 20, and *K* = 45 in this study. As the prior for *α*_*i*_, *β*_*j*_, and *γ*_*k*_, random-walk of first-order was used. *z*_*ij*_ are assumed to be generated from a normal distribution whose mean is zero. To identify each APC effect, the sum of each effect was constrained to zero [13]. The Hamiltonian Monte Carlo method was used to estimate the parameters (http://mc-stan.org/). We applied the Poisson model to the data of 47 prefectures and all of Japan for each type of cancer and sex. Using the estimates of the APC effect, the mortality rate ratios among age groups, periods, and cohorts were then calculated for each prefecture. Moreover, the decreasing rate of cohort effect from the earliest born cohort to the most recently born cohort was calculated for each type of cancer and each prefecture. Furthermore, the estimated mortality rates for each cohort were calculated using the estimates of the cohort effect and the intercept of the Poisson model for the prefectures. The mortality rate ratio of each prefecture compared with all of Japan was then calculated for three cohorts, i.e., those who were born in 1920–1924, 1940–1944, and 1960–1964. Although we calculated the results of 45 cohorts, we showed the results of only 3 cohorts due to space limitations. By calculating the mortality rate ratio of each prefecture compared with all of Japan for the cohorts, we can assess the relative level of the mortality rate of a prefecture for each cohort. All statistical analyses were conducted using R3.5.1 (https://www.R-project.org/).

## Results

Table 1 shows age-standardized mortality rates for each type of cancer in 1999 and 2018 per 100,000 persons among men. The age-standardized mortality rates decreased for all prefectures for stomach, liver, and lung cancer, and those for some prefectures increased for colorectal cancer.

**Table 1:** Age-standardized mortality rates for each type of cancer in 1999 and 2018 among men

	Stomach cancer		Colorectal cancer		Liver cancer		Lung cancer	
	1999	2018	1999	2018	1999	2018	1999	2018
Hokkaido	101.6	54.4	75.9	62.0	71.0	37.7	151.0	121.9
Aomori	129.9	67.8	84.0	89.3	71.5	39.4	148.0	127.1
Iwate	107.6	55.1	79.1	75.4	52.1	37.0	142.4	103.4
Miyagi	115.5	51.4	68.1	49.0	65.6	32.8	143.3	100.5
Akita	160.7	77.0	68.7	67.6	52.9	30.8	137.7	111.3
Yamagata	135.5	65.1	69.4	55.0	66.5	23.8	139.3	98.4
Fukushima	125.1	59.7	71.8	63.9	63.7	33.8	133.9	101.4
Ibaraki	134.1	59.9	67.3	61.2	86.2	32.5	131.9	101.9
Tochigi	145.6	53.3	71.0	63.6	90.0	38.4	127.4	96.9
Gunma	111.9	54.9	63.8	63.3	80.0	33.0	121.9	100.3
Saitama	122.6	58.3	77.0	62.0	80.3	28.3	130.3	99.7
Chiba	122.1	51.9	72.4	56.2	89.2	29.4	131.4	93.7
Tokyo	120.0	50.1	78.2	60.8	94.4	30.1	126.9	98.5
Kanagawa	120.1	51.1	75.9	57.2	87.3	28.7	123.7	92.5
Niigata	144.0	56.0	69.7	59.8	54.5	22.7	141.3	98.9
Toyama	142.4	49.2	65.8	54.1	69.3	23.7	143.3	87.5
Ishikawa	116.3	56.7	70.3	52.9	85.1	27.1	139.4	96.8
Fukui	113.4	49.1	63.6	54.5	72.3	24.5	138.7	95.1
Yamanashi	78.6	36.8	67.5	62.9	125.0	40.6	124.5	94.0
Nagano	95.7	39.0	59.4	51.4	58.1	23.7	96.4	81.6
Gifu	120.7	60.6	65.8	53.9	89.0	26.9	127.4	101.5
Shizuoka	102.9	47.1	63.1	55.1	109.6	30.5	122.7	94.2
Aichi	115.1	56.5	70.1	55.0	92.5	29.2	145.4	100.8
Mie	112.7	55.6	67.0	50.9	85.6	27.0	134.0	93.3
Shiga	108.4	51.4	60.0	44.8	58.2	24.3	150.4	90.6
Kyoto	120.2	52.2	74.9	56.3	98.3	32.8	149.0	100.0
Osaka	126.6	60.9	76.1	58.5	153.2	39.3	156.9	112.7
Hyogo	119.2	55.2	71.9	47.9	126.7	34.2	138.4	98.8
Nara	113.3	51.5	56.0	46.2	117.0	34.4	140.7	96.9
Wakayama	114.5	51.9	60.4	61.0	129.8	40.4	146.7	127.9
Tottori	104.6	58.2	68.3	69.4	107.2	35.1	143.6	99.3
Shimane	103.3	59.3	83.6	39.0	98.6	33.8	126.3	102.1
Okayama	103.4	47.6	53.3	47.5	112.1	38.1	130.2	101.2
Hiroshima	113.4	54.4	63.8	53.6	144.0	46.0	137.3	97.8
Yamaguchi	125.0	60.8	78.3	55.4	121.6	31.5	141.7	101.1
Tokushima	114.7	51.3	52.0	56.4	110.0	42.0	131.6	105.6
Kagawa	121.9	58.6	47.0	42.3	88.3	38.9	128.1	95.0
Ehime	109.5	56.7	54.6	56.2	113.0	40.1	126.4	99.9
Kochi	117.8	67.6	65.1	59.7	104.7	42.4	128.9	93.6
Fukuoka	112.1	52.3	71.1	60.0	167.2	42.9	150.6	104.8
Saga	140.4	64.7	64.5	55.5	132.4	47.6	135.6	102.1
Nagasaki	94.8	48.2	71.7	56.1	118.5	35.2	157.0	110.9
Kumamoto	82.8	35.9	57.5	41.7	107.5	34.8	118.2	94.6
Oita	95.4	45.7	55.7	47.9	113.4	25.8	122.4	93.2
Miyazaki	96.5	51.3	56.8	66.1	68.1	35.7	133.1	96.7
Kagoshima	76.4	33.1	65.3	56.0	101.8	41.9	124.0	100.2
Okinawa	68.9	26.9	50.6	74.5	44.9	26.4	156.8	84.4

Age-standardized mortality rates for stomach, colorectal, liver, and lung cancer per 100,000 persons among men in 1999 and 2018

Table 2 shows age-standardized mortality rates for each type of cancer in 1999 and 2018 per 100,000 persons among women. The age-standardized mortality rates decreased for all prefectures for stomach and liver cancer, and those for some prefectures increased for colorectal and lung cancer.

**Table 2:** Age-standardized mortality rates for each type of cancer in 1999 and 2018 among women

	Stomach cancer		Colorectal cancer		Liver cancer		Lung cancer	
	1999	2018	1999	2018	1999	2018	1999	2018
Hokkaido	35.8	21.6	41.9	36.9	24.1	10.4	41.0	49.4
Aomori	41.9	20.3	48.8	44.3	25.6	12.5	35.3	33.9
Iwate	37.2	21.6	38.7	39.2	22.3	12.2	29.3	27.9
Miyagi	41.9	19.4	46.9	33.2	20.7	11.4	36.4	31.3
Akita	45.5	27.1	51.2	35.1	21.3	12.3	29.1	22.8
Yamagata	46.5	18.2	49.2	31.4	21.9	6.8	36.6	33.2
Fukushima	42.4	19.2	42.8	37.5	27.2	10.4	35.2	27.6
Ibaraki	49.8	19.0	34.4	33.9	22.3	11.0	30.5	29.5
Tochigi	49.5	19.0	40.8	36.3	31.6	10.2	30.5	32.4
Gunma	44.2	18.2	37.1	36.8	24.9	12.2	31.2	30.6
Saitama	44.3	20.5	39.5	35.4	32.5	10.7	37.9	31.9
Chiba	45.2	19.5	40.6	30.0	29.0	8.6	36.1	32.0
Tokyo	44.6	16.9	45.1	31.7	32.2	8.2	42.5	35.3
Kanagawa	46.2	19.5	42.9	36.4	28.2	10.0	40.1	33.8
Niigata	46.5	23.2	35.0	31.1	16.8	6.4	28.4	29.4
Toyama	54.9	18.3	48.4	27.0	24.4	9.2	25.9	23.8
Ishikawa	42.2	19.6	42.9	29.7	33.0	9.2	38.5	38.2
Fukui	37.4	20.3	39.6	23.8	29.7	10.4	32.2	24.1
Yamanashi	32.2	15.0	28.3	28.7	41.0	14.1	32.3	27.9
Nagano	36.0	18.6	38.0	32.7	24.7	11.0	28.6	22.5
Gifu	53.4	26.2	43.9	36.9	27.6	9.8	29.9	32.9
Shizuoka	35.5	18.5	39.6	29.0	26.5	9.7	35.5	31.7
Aichi	48.2	20.6	45.7	34.2	29.0	10.6	38.6	32.0
Mie	54.3	21.3	39.4	33.8	28.8	8.2	33.5	25.6
Shiga	40.8	19.4	38.1	30.1	25.4	9.6	36.3	24.9
Kyoto	41.2	18.4	38.4	32.2	39.1	10.4	41.4	35.6
Osaka	46.2	21.9	44.9	33.5	49.2	12.4	46.9	38.3
Hyogo	46.6	21.4	43.8	33.7	43.3	11.1	41.4	27.8
Nara	50.2	17.6	33.1	32.2	36.3	10.8	40.7	27.7
Wakayama	49.3	16.8	33.5	40.2	36.0	11.6	30.4	30.5
Tottori	46.5	15.2	35.0	29.6	29.5	15.5	42.4	31.3
Shimane	34.9	23.4	45.0	36.1	28.8	10.0	24.4	25.5
Okayama	39.8	16.9	31.7	30.2	37.6	12.4	31.2	25.3
Hiroshima	34.1	17.1	37.5	33.6	40.4	12.4	37.4	28.8
Yamaguchi	40.5	23.1	38.3	39.1	36.9	12.9	42.2	32.9
Tokushima	40.5	18.6	39.3	23.0	28.2	12.5	26.4	31.8
Kagawa	43.6	24.6	31.4	26.1	35.6	7.3	37.5	33.5
Ehime	40.8	24.7	35.6	30.3	36.4	12.4	38.7	27.2
Kochi	28.9	19.1	31.6	24.8	32.7	12.6	31.8	29.9
Fukuoka	42.3	21.2	40.9	34.8	45.7	13.4	44.2	33.3
Saga	38.2	19.1	36.7	30.1	52.3	10.1	34.5	35.1
Nagasaki	34.7	24.0	36.6	42.4	37.0	10.4	41.6	37.2
Kumamoto	27.5	13.5	28.4	29.4	33.3	12.5	35.6	26.1
Oita	36.2	15.7	32.0	26.1	45.2	15.1	38.4	25.6
Miyazaki	27.8	16.8	31.1	26.7	31.5	12.0	27.1	30.2
Kagoshima	34.1	15.4	33.0	26.1	32.8	13.6	37.5	23.8
Okinawa	19.2	11.1	31.2	24.5	14.9	7.4	51.2	26.1

Age-standardized mortality rates for stomach, colorectal, liver, and lung cancer per 100,000 persons among women in 1999 and 2018

**Fig. 1:**
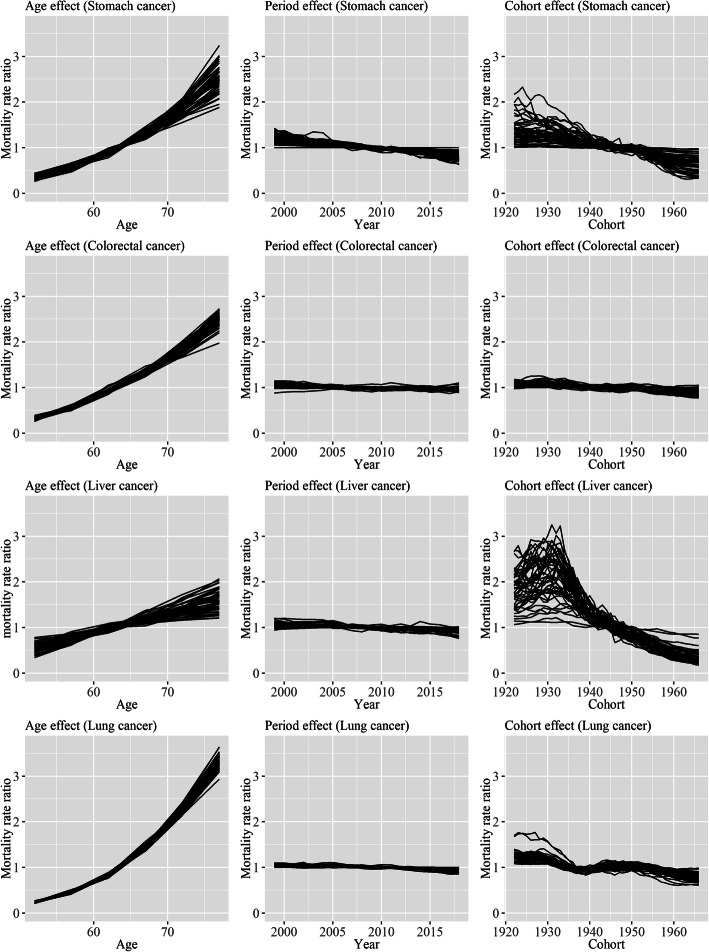
Age, period, and cohort effects of 47 prefectures for each type of cancer in men. Each line signifies point estimates of each effect for each prefecture, and values of time points in an effect are connected for each prefecture. Regarding age effect, the value for each 5-year age group is assigned to the midpoint of each 5-year age group

**Fig. 2:**
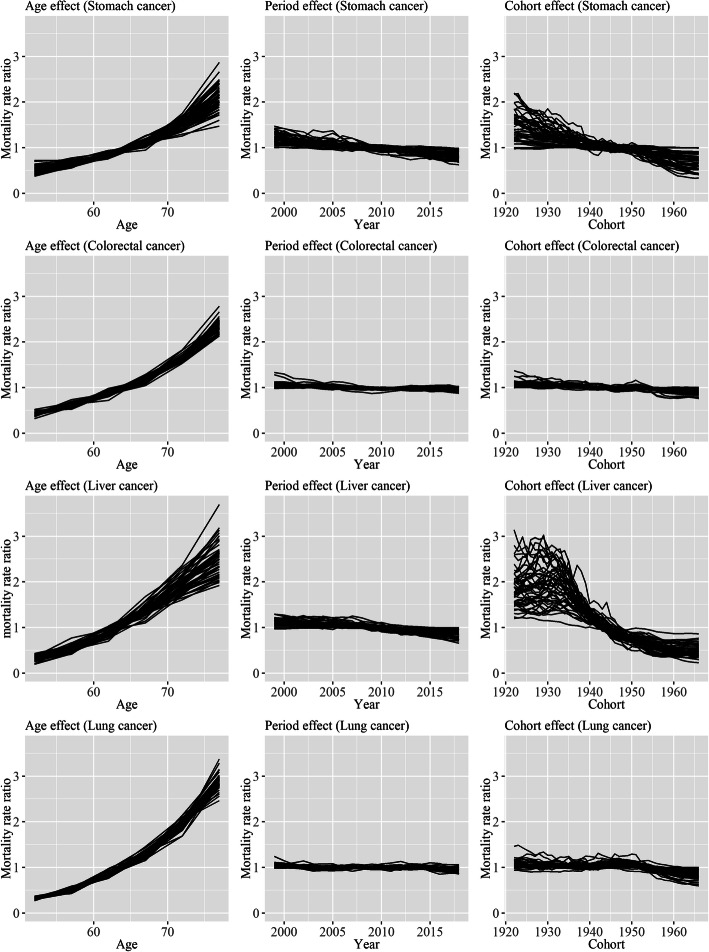
Age, period, and cohort effects of 47 prefectures for each type of cancer in women. Each line signifies point estimates of each effect for each prefecture, and values of time points in an effect are connected for each prefecture. Regarding age effect, the value for each 5-year age group is assigned to the midpoint of each 5-year age group

Figures 1 and 2 show the APC effect of prefectures for each type of cancer in men and women. The age effect increased in all prefectures for all types of cancer. Period effects showed slight decreasing trends overall for all types of cancer, and there was little difference in the trends among prefectures. The decreasing rates of cohort effects were largely different among prefecture for stomach and liver cancer, particularly for liver cancer. On the other hand, the differences in the decreasing rates of cohort effects were smaller for colorectal and liver cancer. The decreasing rates of period effects were relatively small, and a large part of the decreasing rates of age-standardized mortality rates of stomach and liver cancer was caused by the decreasing rates of the cohort effects.

Table 3 shows the decreasing rates of cohort effects in cohorts born between 1920 and 1924 and 1964 and 1968 for each type of cancer and the ranks of the decreasing rates among prefectures in men and women. The ranks of the decreasing rates for a prefecture varied depending on the type of cancer. However, the decreasing rates of cohort effect did not tend to be lower for prefectures that include metropolitan areas, such as Chiba, Tokyo, Kanagawa, Aichi, Kyoto, Osaka, Hyogo, and Fukuoka, regardless of cancer type.

**Table 3:** Decreasing rates of cohort effects among prefectures and the ranks of the decreasing rates

Prefecture	Men				Women			
	Stomach cancer	Colorectal cancer	Liver cancer	Lung cancer	Stomach cancer	Colorectal cancer	Liver cancer	Lung cancer
Hokkaido	4.9 (46)	− 0.0 (44)	78.1 (34)	30.3 (33)	27.2 (38)	8.2 (35)	68.3 (30)	− 0.7 (47)
Aomori	36.7 (36)	0.1 (42)	20.2 (47)	20.2 (46)	32.2 (35)	10.5 (31)	44.1 (46)	9.8 (36)
Iwate	83.3 (2)	5.9 (34)	49.3 (45)	30.0 (34)	58.8 (22)	3.9 (46)	57.6 (41)	7.1 (40)
Miyagi	43.0 (30)	11.3 (25)	60.4 (44)	35.5 (23)	46.9 (27)	16.3 (15)	55.6 (43)	11.2 (34)
Akita	64.1 (13)	15.8 (19)	67.2 (41)	21.1 (45)	63.5 (17)	10.9 (28)	59.5 (38)	12.4 (31)
Yamagata	10.0 (45)	11.3 (24)	83.5 (23)	44.0 (9)	0.0 (45)	10.7 (29)	59.5 (39)	14.7 (28)
Fukushima	57.3 (17)	15.0 (20)	66.2 (42)	34.9 (24)	20.4 (42)	8.8 (34)	74.7 (24)	19.8 (22)
Ibaraki	29.4 (39)	0.9 (41)	80.9 (30)	32.2 (29)	64.7 (15)	1.7 (47)	69.6 (29)	9.8 (35)
Tochigi	48.0 (25)	3.3 (39)	72.5 (37)	26.7 (40)	67.8 (11)	7.6 (37)	79.1 (15)	7.4 (39)
Gunma	68.5 (9)	− 1.1 (47)	81.7 (27)	29.7 (35)	0.4 (44)	5.8 (43)	64.0 (35)	20.8 (20)
Saitama	42.0 (33)	16.4 (15)	69.4 (38)	31.3 (30)	80.4 (2)	10.7 (30)	76.5 (22)	34.8 (8)
Chiba	64.2 (12)	27.7 (5)	82.2 (25)	39.3 (14)	43.4 (28)	11.4 (25)	77.4 (19)	22.9 (18)
Tokyo	82.0 (3)	28.6 (3)	88.7 (7)	48.1 (6)	66.2 (13)	19.0 (11)	76.9 (20)	26.0 (16)
Kanagawa	65.7 (11)	27.7 (4)	89.2 (5)	34.2 (25)	70.7 (9)	6.5 (40)	78.4 (16)	26.9 (14)
Niigata	49.2 (22)	8.3 (31)	80.7 (32)	39.1 (16)	31.2 (37)	15.4 (16)	75.8 (23)	8.8 (37)
Toyama	17.2 (43)	12.7 (23)	84.6 (20)	37.5 (18)	26.0 (39)	49.0 (1)	65.3 (33)	27.9 (12)
Ishikawa	84.0 (1)	20.0 (12)	79.2 (33)	31.0 (32)	31.4 (36)	24.3 (5)	71.5 (27)	37.9 (5)
Fukui	47.2 (26)	10.7 (27)	83.7 (22)	29.0 (37)	40.7 (30)	22.1 (8)	45.3 (45)	44.9 (3)
Yamanashi	48.3 (24)	0.0 (43)	88.8 (6)	27.7 (38)	49.2 (25)	6.4 (41)	85.0 (5)	13.0 (30)
Nagano	26.2 (41)	7.0 (32)	68.5 (40)	36.2 (20)	− 1.7 (47)	13.3 (23)	71.3 (28)	12.3 (32)
Gifu	50.5 (21)	13.7 (22)	86.4 (14)	27.0 (39)	15.3 (43)	17.2 (14)	68.1 (31)	18.7 (24)
Shizuoka	78.1 (6)	10.5 (28)	86.3 (16)	38.6 (17)	76.4 (4)	5.9 (42)	77.8 (18)	11.6 (33)
Aichi	78.5 (5)	28.8 (2)	85.7 (17)	42.3 (10)	67.6 (12)	39.9 (2)	84.2 (6)	32.2 (9)
Mie	48.9 (23)	25.3 (7)	88.0 (11)	32.8 (28)	71.9 (8)	17.4 (13)	87.0 (4)	35.3 (7)
Shiga	42.5 (32)	21.1 (11)	80.8 (31)	62.5 (2)	79.2 (3)	22.8 (6)	87.8 (2)	15.6 (26)
Kyoto	81.8 (4)	19.1 (13)	82.0 (26)	41.4 (11)	84.6 (1)	13.4 (22)	84.1 (8)	48.8 (2)
Osaka	74.4 (7)	29.2 (1)	89.9 (3)	49.8 (3)	74.7 (5)	20.1 (9)	88.6 (1)	27.7 (13)
Hyogo	69.8 (8)	21.3 (10)	91.8 (2)	37.4 (19)	65.4 (14)	18.3 (12)	82.1 (11)	23.9 (17)
Nara	35.9 (37)	16.2 (16)	87.1 (12)	22.6 (43)	49.6 (24)	13.5 (21)	87.0 (3)	6.0 (43)
Wakayama	4.1 (47)	5.0 (36)	84.8 (19)	19.8 (47)	61.1 (19)	14.8 (17)	82.6 (10)	8.6 (38)
Tottori	28.1 (40)	9.9 (29)	86.3 (15)	22.2 (44)	74.0 (7)	13.1 (24)	58.5 (40)	38.3 (4)
Shimane	45.2 (28)	26.8 (6)	88.7 (8)	24.5 (41)	36.1 (32)	27.0 (4)	74.0 (25)	6.4 (42)
Okayama	53.9 (19)	21.4 (9)	84.3 (21)	33.8 (26)	74.0 (6)	14.8 (18)	76.9 (21)	16.2 (25)
Hiroshima	59.6 (16)	14.1 (21)	89.8 (4)	44.9 (8)	60.2 (21)	5.7 (45)	84.2 (7)	15.0 (27)
Yamaguchi	43.4 (29)	8.9 (30)	81.2 (29)	48.3 (5)	24.1 (40)	6.5 (39)	81.9 (12)	21.7 (19)
Tokushima	61.3 (15)	2.2 (40)	88.5 (10)	40.9 (12)	63.4 (18)	36.0 (3)	71.7 (26)	4.4 (44)
Kagawa	61.9 (14)	6.5 (33)	76.2 (35)	32.9 (27)	70.6 (10)	7.8 (36)	78.3 (17)	6.8 (41)
Ehime	53.1 (20)	5.5 (35)	85.7 (18)	31.3 (31)	60.3 (20)	5.8 (44)	54.8 (44)	55.0 (1)
Kochi	41.8 (34)	3.6 (38)	68.9 (39)	29.2 (36)	− 0.1 (46)	22.4 (7)	66.9 (32)	13.7 (29)
Fukuoka	68.4 (10)	15.9 (17)	92.4 (1)	35.6 (22)	35.5 (33)	19.5 (10)	56.1 (42)	31.6 (10)
Saga	46.4 (27)	24.3 (8)	88.6 (9)	23.3 (42)	64.6 (16)	9.4 (32)	80.4 (13)	3.6 (45)
Nagasaki	35.8 (38)	16.9 (14)	86.9 (13)	48.9 (4)	21.6 (41)	7.2 (38)	80.2 (14)	19.8 (21)
Kumamoto	41.6 (35)	4.4 (37)	82.6 (24)	39.9 (13)	34.6 (34)	9.0 (33)	63.0 (36)	28.6 (11)
Oita	43.0 (31)	15.9 (18)	75.0 (36)	39.1 (15)	54.0 (23)	11.2 (26)	82.8 (9)	18.9 (23)
Miyazaki	17.6 (42)	− 0.3 (45)	60.5 (43)	45.6 (7)	39.2 (31)	13.6 (20)	64.6 (34)	0.0 (46)
Kagoshima	13.7 (44)	11.2 (26)	81.4 (28)	36.1 (21)	47.2 (26)	13.7 (19)	62.7 (37)	35.7 (6)
Okinawa	54.5 (18)	− 0.6 (46)	32.4 (46)	64.1 (1)	41.6 (29)	11.1 (27)	28.0 (47)	26.6 (15)

The decreasing rates of cohort effects in cohorts born between 1920 and 1924 and between 1964 and 1968 for stomach, colorectal, liver, and lung cancer and the ranks of the decreasing rates among prefectures in men and women. The decreasing rates are displayed as a percentage. The values in parentheses indicate the rank of the decreasing rate among prefectures

*The value in parentheses is the rank of the decreasing rate among prefectures

Tables 4 and 5 show the relative mortality rate of a prefecture compared with all of Japan for cohorts born in 1920–1924, 1940–1944, and 1960–1964 for each type of cancer in men and women. The relative mortality rates varied widely depending on cohorts, particularly for stomach and liver cancer.

**Table 4:** Mortality rate ratio of a prefecture to all of Japan on each cohort in men

	Stomach cancer			Colorectal cancer			Liver cancer			Lung cancer		
	1920–1924	1940–1944	1960–1964	1920–1924	1940–1944	1960–1964	1920–1924	1940–1944	1960–1964	1920–1924	1940–1944	1960–1964
Hokkaido	0.954	0.939	1.392	1.026	1.040	1.201	0.669	0.998	1.162	1.068	1.202	1.267
Aomori	1.406	1.253	1.449	1.342	1.344	1.596	0.559	0.989	2.932	1.099	1.199	1.355
Iwate	1.787	1.044	0.445	1.105	1.153	1.250	0.451	0.792	1.633	1.093	0.953	1.120
Miyagi	1.203	0.992	1.088	0.973	0.928	1.034	0.454	0.755	1.324	1.022	1.022	1.019
Akita	1.982	1.498	1.171	1.343	1.161	1.333	0.484	0.825	1.174	1.099	0.962	1.279
Yamagata	1.213	1.193	1.681	1.032	0.948	1.073	0.582	0.651	0.755	1.066	0.959	0.938
Fukushima	1.525	1.114	1.039	1.156	1.054	1.161	0.592	0.812	1.378	0.999	0.953	1.004
Ibaraki	1.273	1.072	1.331	1.045	1.004	1.195	0.656	0.852	1.128	0.978	0.957	1.034
Tochigi	1.397	1.177	1.193	1.078	1.068	1.220	0.737	1.019	1.344	0.983	0.934	1.071
Gunma	1.484	1.100	0.776	1.011	1.032	1.191	0.697	0.941	1.143	0.859	0.908	0.954
Saitama	1.042	1.044	1.030	1.089	1.047	1.078	0.581	0.780	1.454	0.909	0.959	1.006
Chiba	1.314	1.016	0.817	1.130	0.976	0.989	0.755	0.815	1.108	0.990	0.968	0.946
Tokyo	1.725	1.084	0.578	1.143	1.122	1.006	0.913	0.938	0.905	1.017	1.028	0.804
Kanagawa	1.305	1.011	0.771	1.115	1.036	0.970	0.919	0.845	0.889	0.860	0.969	0.893
Niigata	1.428	1.213	1.167	1.033	1.001	1.111	0.556	0.674	0.841	1.138	1.002	1.048
Toyama	1.156	1.074	1.471	0.985	0.942	0.978	0.857	0.762	0.927	0.950	0.899	0.939
Ishikawa	2.079	1.127	0.537	1.010	0.923	0.972	0.764	0.836	1.184	1.045	0.992	1.101
Fukui	1.162	0.883	0.988	0.962	0.863	0.997	0.753	0.910	0.900	0.995	0.920	1.073
Yamanashi	1.151	0.800	0.866	0.933	0.946	1.093	1.045	1.295	0.928	0.858	0.811	0.939
Nagano	0.850	0.762	0.963	0.879	0.886	0.965	0.453	0.733	1.039	0.733	0.773	0.712
Gifu	1.292	1.016	1.046	0.997	0.972	1.007	0.863	0.953	0.916	0.960	0.935	1.093
Shizuoka	1.608	0.881	0.578	0.954	0.930	1.011	0.972	0.878	1.141	0.897	0.917	0.863
Aichi	1.660	1.078	0.646	1.055	1.007	0.923	0.807	0.841	0.952	1.039	1.052	1.013
Mie	1.241	0.884	0.989	1.012	0.894	0.910	0.761	0.920	0.706	0.963	1.025	1.008
Shiga	1.174	0.974	1.027	0.948	0.862	0.857	0.565	0.746	0.891	1.419	1.087	0.782
Kyoto	1.675	0.994	0.608	1.048	0.991	1.004	0.867	0.987	1.112	1.053	1.000	0.992
Osaka	1.824	1.176	0.737	1.149	1.062	0.988	1.715	1.252	1.280	1.255	1.145	1.044
Hyogo	1.499	1.057	0.724	1.013	0.960	0.963	1.455	1.218	0.878	1.060	1.053	1.066
Nara	1.294	1.115	1.327	0.996	0.825	0.957	1.114	0.895	1.302	0.979	0.966	1.178
Wakayama	1.103	1.075	1.602	1.052	1.055	1.183	1.239	1.323	1.401	1.115	1.173	1.359
Tottori	1.278	1.165	1.416	0.994	1.086	1.101	1.089	1.410	1.077	1.056	1.029	1.303
Shimane	1.185	0.995	1.017	1.060	1.033	0.928	1.145	1.323	0.921	1.044	0.988	1.159
Okayama	1.163	0.929	0.877	0.878	0.867	0.803	1.112	1.093	1.141	1.023	0.983	1.020
Hiroshima	1.290	1.004	0.857	0.985	0.881	1.005	1.768	1.448	1.326	1.102	1.009	0.934
Yamaguchi	1.334	1.061	1.164	1.083	1.069	1.165	1.137	1.208	1.556	1.140	1.002	0.947
Tokushima	1.447	0.989	0.853	0.920	0.920	1.048	1.178	1.284	0.974	1.046	1.025	0.965
Kagawa	1.480	1.023	0.909	0.801	0.799	0.880	0.882	1.051	1.615	1.018	0.988	1.026
Ehime	1.320	1.104	0.986	0.888	0.840	0.977	1.433	1.350	1.311	1.046	0.985	1.122
Kochi	1.296	1.032	1.165	1.052	0.906	1.174	0.811	1.176	1.743	0.978	1.000	1.014
Fukuoka	1.457	1.020	0.758	1.096	1.022	1.106	1.630	1.604	1.186	1.094	1.030	1.061
Saga	1.350	1.133	1.167	1.084	1.027	0.953	1.342	1.674	1.307	1.024	1.013	1.195
Nagasaki	1.069	0.905	1.070	1.218	0.979	1.187	1.223	1.342	1.228	1.304	1.128	1.066
Kumamoto	0.761	0.629	0.689	0.823	0.825	0.931	1.032	1.293	1.510	0.922	0.901	0.940
Oita	1.033	0.812	0.901	0.883	0.838	0.852	0.906	1.216	1.782	1.073	0.933	1.016
Miyazaki	0.937	0.854	1.172	0.838	0.863	0.993	0.651	1.054	1.839	1.094	0.911	0.944
Kagoshima	0.741	0.707	0.997	0.951	0.997	1.019	0.872	1.258	1.182	1.054	0.920	0.999
Okinawa	0.853	0.544	0.627	1.176	1.195	1.385	0.377	0.628	1.825	1.339	0.844	0.743

The mortality rate ratio of a prefecture to all of Japan on the cohorts born in 1920–1924, 1940–1944, and 1960–1964 for stomach, colorectal, liver, and lung cancer in men

**Table 5:** Mortality rate ratio of a prefecture to all of Japan on each cohort in women

	Stomach cancer			Colorectal cancer			Liver cancer			Lung cancer		
	1920–1924	1940–1944	1960–1964	1920–1924	1940–1944	1960–1964	1920–1924	1940–1944	1960–1964	1920–1924	1940–1944	1960–1964
Hokkaido	0.893	0.917	1.248	1.052	0.992	0.981	1.058	0.951	0.866	1.111	1.211	1.261
Aomori	1.161	1.073	1.445	1.241	1.137	1.148	0.980	1.032	1.296	0.976	0.892	0.966
Iwate	1.160	0.956	0.898	0.991	1.008	0.971	0.961	0.848	0.998	0.799	0.756	0.796
Miyagi	1.026	0.928	1.005	1.023	0.947	0.865	0.936	0.813	0.975	0.912	0.853	0.861
Akita	1.590	1.230	1.169	1.071	1.013	0.983	0.798	0.739	0.824	0.805	0.819	0.761
Yamagata	0.852	1.067	1.556	0.912	0.870	0.830	0.879	0.659	0.850	0.844	0.826	0.776
Fukushima	0.861	1.012	1.303	0.974	0.922	0.885	1.315	0.889	0.866	0.859	0.893	0.762
Ibaraki	1.291	1.118	0.903	0.923	0.908	0.929	1.142	0.842	0.847	0.880	0.882	0.873
Tochigi	1.356	1.139	0.915	1.043	0.941	0.985	1.419	0.956	0.743	0.820	0.886	0.799
Gunma	0.751	0.941	1.366	0.916	0.915	0.886	1.074	1.013	0.950	0.894	1.057	0.670
Saitama	1.851	1.160	0.655	1.017	0.914	0.921	1.254	0.803	0.678	0.975	0.992	0.681
Chiba	1.018	1.009	1.106	0.930	0.896	0.867	1.219	0.850	0.710	0.951	0.928	0.794
Tokyo	1.124	1.010	0.750	1.070	0.958	0.898	1.310	0.878	0.754	1.061	0.991	0.874
Kanagawa	1.391	1.021	0.771	1.019	0.987	0.979	1.283	0.836	0.700	1.066	0.948	0.862
Niigata	0.999	1.113	1.280	0.939	0.896	0.805	0.811	0.745	0.515	0.792	0.749	0.784
Toyama	1.038	1.137	1.427	1.291	0.934	0.696	1.044	0.744	0.982	0.760	0.706	0.602
Ishikawa	0.934	1.086	1.207	0.987	0.896	0.785	1.333	1.011	0.975	0.891	0.896	0.633
Fukui	1.053	0.999	1.169	0.895	0.827	0.756	1.109	1.072	1.414	0.887	0.786	0.556
Yamanashi	1.000	0.852	0.984	0.804	0.790	0.768	2.647	1.189	0.838	0.831	0.778	0.792
Nagano	0.634	0.788	1.186	0.926	0.796	0.843	0.981	0.853	0.669	0.771	0.689	0.719
Gifu	1.037	1.211	1.611	1.079	0.997	0.942	1.151	0.982	0.868	0.829	0.908	0.751
Shizuoka	1.228	1.022	0.667	0.888	0.867	0.855	1.342	0.943	0.686	0.842	0.854	0.801
Aichi	1.418	1.097	0.955	1.233	0.942	0.773	1.638	0.963	0.703	1.126	0.993	0.846
Mie	1.468	1.064	0.824	0.937	0.864	0.794	1.603	0.795	0.514	0.985	0.877	0.725
Shiga	1.627	1.088	0.736	0.928	0.792	0.746	1.021	1.031	0.369	0.930	0.845	0.833
Kyoto	1.598	1.007	0.498	1.021	0.981	0.895	2.049	1.198	0.729	1.153	1.050	0.702
Osaka	1.526	1.113	0.877	1.035	0.975	0.859	2.431	1.242	0.803	1.211	1.127	0.978
Hyogo	1.253	1.075	0.877	1.019	0.931	0.865	1.823	1.258	0.921	1.029	0.952	0.877
Nara	1.113	1.084	1.059	0.869	0.793	0.755	2.130	1.057	0.726	1.008	0.950	1.008
Wakayama	1.368	1.215	1.031	1.014	1.036	0.877	1.714	1.372	0.739	0.931	0.933	0.931
Tottori	1.849	0.965	0.950	0.973	0.987	0.875	1.204	1.134	1.215	1.001	1.012	0.673
Shimane	0.995	1.112	1.144	0.995	0.989	0.751	1.450	1.253	0.989	0.730	0.737	0.736
Okayama	1.222	1.028	0.689	0.770	0.743	0.702	1.572	1.016	0.936	0.797	0.749	0.722
Hiroshima	1.133	0.956	0.890	0.897	0.852	0.870	1.991	1.430	0.763	0.935	0.855	0.853
Yamaguchi	1.005	1.104	1.406	0.900	0.943	0.855	1.887	1.156	0.899	1.025	0.931	0.872
Tokushima	1.236	1.096	0.830	0.943	0.825	0.627	1.416	1.308	0.992	0.808	0.757	0.820
Kagawa	1.853	1.063	0.968	0.789	0.766	0.743	1.269	1.130	0.696	0.889	0.831	0.893
Ehime	1.278	1.030	1.027	0.803	0.792	0.784	1.225	1.207	1.370	1.203	0.781	0.559
Kochi	0.772	0.981	1.413	0.887	0.778	0.711	1.272	1.279	1.084	0.961	0.838	0.864
Fukuoka	0.939	0.963	1.146	1.039	0.984	0.863	1.602	1.447	1.635	1.179	1.016	0.853
Saga	1.524	1.058	1.029	0.960	0.903	0.882	2.648	1.731	1.190	0.900	0.854	0.928
Nagasaki	0.906	0.963	1.316	1.051	0.942	0.986	1.617	1.317	0.856	1.070	0.897	0.921
Kumamoto	0.697	0.713	0.870	0.781	0.756	0.733	1.241	1.193	1.127	1.006	0.837	0.801
Oita	1.010	0.823	0.872	0.790	0.756	0.731	1.877	1.349	0.848	0.931	0.838	0.826
Miyazaki	0.823	0.845	0.930	0.854	0.776	0.776	1.227	1.215	1.060	0.831	0.802	0.883
Kagoshima	0.821	0.745	0.872	0.891	0.763	0.793	0.932	1.216	0.929	1.005	0.855	0.708
Okinawa	0.534	0.568	0.567	0.873	0.855	0.794	0.604	0.611	1.061	1.018	0.812	0.813

The mortality rate ratio of a prefecture to all of Japan on the cohorts born in 1920–1924, 1940–1944, and 1960 –1964 for stomach, colorectal, liver, and lung cancer in women

## Discussion

With regard to stomach cancer, there was a large difference in the decreasing rates of the cohort effect among prefectures. The decrease of stomach cancer mortality is one of the largest factors for the decrease of all-sites cancer in Japan [2], and it is possible that the difference in the decreasing rates of cohort effects among prefectures is related to the difference in the decreasing rates of all-sites cancer throughout the years. The decreasing rate of the cohort effect tended to be relatively large in prefectures that have metropolitan cities. The largest cause of the decrease of the cohort effect for stomach cancer mortality rate is believed to be the decrease of prevalence of *Helicobacter pylori* (*H. pylori*) [7, 9]. It is known that the prevalence of *H. pylori* is decreasing among cohorts in Japan [14]. Socioeconomic status (SES) is considered to be associated with the degree of the decrease of the infection in other countries [15]. For example, in China, it was shown that having a lower family income and lower education level are significant risk factors associated with *H. pylori* infection in rural areas [16]. A decrease of the prevalence of *H. pylori* infection was shown to be associated with urbanization [17], and it is believed that sanitary conditions, such as contaminated food and water, are related to the infection [18, 19]. Therefore, it is considered that the prevalence of *H. pylori* infection especially decreased in prefectures with a large decreasing rate of the cohort effect and that there is a difference in the decreasing rates of the infection among prefectures.

Although the decrease of the cohort effect was observed in most of the prefectures, the decreasing rates were relatively low for colorectal cancer than the other cancer types. However, there are prefectures where a decrease of the cohort effect was not observed for men, and the reason for this should be considered. Although the age-standardized mortality rate of colorectal cancer increased over the years in the late twentieth century, the age-standardized incidence rate of colorectal cancer began to decrease in the 1990s [2]. The increased age-standardized incidence rate of colorectal cancer until 1990 is said to be the result of an increased Westernization of Japanese diets [7, 20], and changes in dietary habits or obesity prevalence across cohorts are considered to be different among prefectures. On the other hand, cohort effects on obesity prevalence were shown to be stable during the analyzed cohorts for men [21], and fat intake was shown to have increased during the analyzed cohorts. Therefore, other factors in addition to changes in dietary habits or prevalence of obesity are considered to also have affected the difference in the decreasing rates of the cohort effect. As another factor, the introduction of colorectal cancer screening is considered to be associated with the decrease in the incidence and mortality of colorectal cancer [22, 23]. In the USA and Korea, introduction of the screening or colonoscopy is considered to have affected the trend of cohort effect on the mortality of colorectal cancer [23, 24], and these factors might also have affected differences in trends of cohort effects in Japan.

The difference in the decreasing rates of cohort effects among prefectures for liver cancer was the largest among the analyzed cancer types. The largest factor for the decrease of the cohort effect is considered to be the decrease of hepatitis C virus over the cohorts [7], because liver cancer is mainly caused by hepatitis C virus in Japan [7, 25, 26]. Hepatitis C infection generally occurs through blood transfusion, and a decrease of infusion with contaminated blood resulting from blood transfusion screening is considered to have contributed the decrease of the prevalence of hepatitis C virus [25]. It is possible that the advances in blood transfusion screening account for some of the difference in the decreasing cancer rates of the cohort effect among the prefectures. Globally, hepatitis C virus infection caused by blood injection is a worldwide problem, particularly for developing countries [27, 28], and SES is considered to be associated with the decreasing rates of the cohort effect.

With regard to lung cancer, the decreases of cohort effect were observed in most of the prefectures, and the differences among prefectures were relatively large for women. Smoking prevalence is generally considered to be associated with the trend of lung cancer [29, 30], and association between smoking prevalence and lung cancer mortality among prefectures has been shown in Japan [31]. However, the cigarette consumption by Japanese people increased during the era of rapid economic growth era (e.g., the 1950s to the 1970s) [32], and smoking prevalence is considered to have increased among the more recent cohorts. Therefore, other factors in addition to smoking prevalence are considered to also have contributed to the differences among prefectures. This possibility was also pointed out in a previous study analyzing the trend of lung cancer for multiple countries [33]. Amelioration of hygiene and public health have been suggested as the reason for the decrease of the cohort effect in Japan [8], and these factors might also help to explain the observed change in the cohort effects.

This study has several limitations. First, although we used the data of individuals aged 50 to 79 years, the data of those 80 years old and older could not be used because population data for the older ages could not be obtained. As the mortality rate of cancer is higher in older ages, the precision of our estimates would be improved if data of older individuals could be used. Also, the mortality of those aged 50 to 54 years was low, depending on cancer type and prefecture, and the precision of the estimates of the recently born cohorts might be lower, particularly for women. Furthermore, our data was limited to the time spanning from 1999 to 2018. This period is relatively short, and it might have affected the estimates of birth cohorts with limited data. It would be meaningful to verify our analysis results using data from more prolonged periods of time. Moreover, the variability of the change in age effects was also large for stomach and liver cancer. We found that prefectures with a large decreasing rate of the cohort effect tend to have a smaller increasing rate of the age effect for liver cancer and stomach cancer in the analysis. Although there is a possibility that the age and cohort effects were not properly identified in this case, we confirmed the convergence of parameters for each age, period, and cohort effect based on R-hat (an index of convergence of parameters), and the parameters were properly identified. However, there is a possibility that it is difficult to accurately reveal the age and cohort effects for prefectures from the data of limited periods. Finally, we need to note that birth cohorts in a prefecture can change based on immigrations and emigrations among prefectures. Therefore, a birth cohort in a prefecture is not same throughout the analyzed periods, particularly in a prefecture that has larger immigration, such as Tokyo. On the other hand, the strength of this study is that we used the Vital Statistics in Japan, and therefore, the results of this study are generalizable to all of Japan.

Finally, the results of this study indicate that the relative risk for cancer in each prefecture differed depending on the type of cancer and birth cohort. Therefore, the results of this study would be a useful resource for discussing preventive measures against each type of cancer for each prefecture. The decrease of age-standardized mortality rates of Japan is a result of the decrease of age-standardized mortality rates for stomach and liver cancer, and it was shown from this study that decreasing rates of cohort effects varied among prefectures. Therefore, there is a possibility this will influence the cancer mortality rates in each prefecture in the future. Each prefecture needs to take preventive measures such as further recommendations for cancer screenings, particularly if the mortality rate of specific cancers increases over cohorts within a specific prefecture. We discussed various possible factors for the trends of cohort effects for each type of cancer. We also discussed the decreasing rate of prevalence of *H. pylori* or hepatitis C virus infection among prefectures as a possible reason for the difference regarding stomach and liver cancer. An epidemiological study investigating the difference in the prevalence among prefectures over the cohorts will be useful for verifying our hypothesis. If there are actually large differences in prevalence trends among prefectures, intensive examination and care are needed for the residents of prefectures with high prevalence. Regarding colorectal cancer, introduction of the screening and colonoscopy is believed to have affected the trend of cohort effects in other countries, and analyzing the trend of the screening rate over cohorts for each prefecture will be meaningful for future research. Also, although the decreasing rates of cohort effect varied depending on cancer type for each prefecture, the decreasing rates of cohort effect did not tend to be lower for prefectures with large metropolitan areas, regardless of cancer types. It is known that SES is associated with many types of cancer [34], and some studies have indicated a difference in the trends of cohort effect depending on SES [35, 36]. It is possible that the decreasing rate of cohort effect varied depending on SES not only for stomach and liver cancer but also for colorectal and lung cancer. As one possible factor for this phenomenon, it is known that smoking prevalence is related to many types of cancer [37–40], and the decreasing rates of smoking prevalence are related to SES [41, 42]. Therefore, there is a possibility that not only sanitary environments but also changes in lifestyle are possibly related to the change of cohort effects for each type of cancer. For understanding the relationship between SES and the decreasing rates of cancer, epidemiological studies focusing on differences in the trend of cohort effect among individual or regional SES will be useful in the future.

## Conclusions

As a result of APC analysis, there was a large difference in the decreasing rates of cohort effects for stomach and liver cancer mortality rates among prefectures for both men and women. However, the differences among prefectures for colorectal and lung cancer were relatively small. The decrease of age-standardized mortality rates for stomach and liver cancer was mainly caused by a decrease in cohort effects. The difference in the decreasing rates of cohort effect might influence the cancer mortality rates of each prefecture in the future.

## Data Availability

The data used in this study can be obtained from the website of Japanese government statistics.

## Abbreviations

APC: Age-period-cohort
SES: Socioeconomic status
*H. pylori*: *Helicobacter pylori*
